# The Relationship Between Population Attributable Fraction and Heritability in Genetic Studies

**DOI:** 10.3389/fgene.2018.00352

**Published:** 2018-10-01

**Authors:** Tao Wang, H. Dean Hosgood, Qing Lan, Xiaonan Xue

**Affiliations:** Department of Epidemiology and Population Health, Albert Einstein College of Medicine, The Bronx, NY, United States

**Keywords:** population attributable risk, heritability, GWAS, summary statistics, genetic epidemiology

## Abstract

Population attributable fraction (PAF) has been widely used to quantify the proportion of disease risk in a population that can be attributed to risk factors in epidemiological studies. However, the use of PAF has been limited in assessing the contribution of genetic variants. Most notably, the PAF estimate is typically much larger than other commonly used measures, such as heritability, thereby raising the concern that PAF may overestimate the genetic contribution. In this paper, we show that PAF is a one-to-one function of heritability, and explain why PAF is larger than heritability. Further, we present an estimation procedure based on the summary statistics from genome-wide association studies (GWAS) to estimate the PAF of multiple correlated genetic variants for a binary outcome. Currently available estimation procedures only apply to a single variant or to multiple genetic variants that are independent from each other. Our simulation studies verified the relationship between PAF and heritability, and showed that the proposed estimation procedure for these two measures performed well. Finally, we applied the proposed method to the published data of two lung cancer GWAS to estimate the PAF and heritability of several newly identified variants. Our results demonstrate that the PAF estimate is a useful measure of the genetic contribution to the development of the disease. We hope this paper serves as an advocate for a wider use of PAF in genetic studies.

## Introduction

Population attributable fraction (PAF) is defined as the reduction in average disease risk by eliminating the exposure(s) of interest from the population, while the other risk factors in the population remain unchanged. Since its introduction by Levin (Levin, 1953), the PAF has been widely used to quantify the proportion of disease risk in a population that can be attributed to a risk factor or a set of risk factors in epidemiological studies (Rockhill et al., 1998; Lim et al., 2012; Burnett et al., 2014; Flegal et al., 2015). When a risk factor is a genetic risk allele, the PAF infers the proportion of disease that is “explained" by this allele (Moonesinghe et al., 2012). Several concerns have been raised for the use of PAF as a measure to assess the contribution of genetic variants to a disease. The primary concern is that the PAF may overestimate the genetic contribution because its estimate is typically much larger than other measures, such as heritability, sibling recurrence risk, and the proportion of area under the curve (Witte et al., 2014). In addition, heritability is considered to be more meaningful because it explains an individual’s “genetic variation in risk."

Heritability is the most commonly used measure of genetic contribution to the risk of a disease, which quantifies the effects on the variability of risk at the population level. On the other hand, PAF measures the effects from genetic variants on the mean level of risk, i.e., the proportion of disease that can be potentially prevented if effective interventions are available. As such, heritability and PAF measure the different aspects of genetic contribution. However, the relationship between them has not been examined. In this paper, we aim to link the PAF estimate and the heritability estimate. Specifically, we first establish a one-to-one function between the PAF and heritability on the observed scale (denoted as *h*^2^ in this paper), which explains why the PAF estimate is often larger than *h*^2^. As *h*^2^ is dependent on the prevalence of the disease in the population, it is often transformed to the underlying liability scale (denoted as $h_{L}^{2}$ in this paper) under the assumption of a classical liability threshold model (Falconer, 1965). Therefore, we show that the one-to-one relationship is applied to $h_{L}^{2}$ as well.

In order to estimate the PAF associated with a genetic variant, information on the allele frequency and its association with disease risk is required. Since such information is routinely available in the summary results from genome-wide association studies (GWAS), the PAF of a single genetic variant can be estimated without making use of the individual genetic data (Witte et al., 2014). For a case-control study, however, to our knowledge there is no existing method to estimate the PAF for a set of correlated variants, because this requires joint effect estimates of the correlated genetic variants from summary statistics. In this paper, we also propose an empirical approach to estimate the PAF using the summary statistics of GWAS case-control studies.

The paper is organized as follows: first, the PAF is defined for both a single variant and multiple genetic variants. Then, its relationship with *h^2^* as well as $h_{L}^{2}$ is derived. Next, an estimation procedure is developed to estimate the PAF using the summary statistics obtained from the case-control GWAS. Subsequently, the established relationship between PAF and heritability and the performance of the estimation procedure for PAF are examined by simulation studies. Finally, the proposed estimation method is applied to estimate the PAF, *h*^2^, and $h_{L}^{2}$ of newly identified genetic variants for lung cancer using the results from the two GWAS conducted in Asian populations.

## Materials and Methods

### Definition of PAF for a Binary Trait Attributed to a Single Variant or Multiple Variants

The population attributable fraction due to a risk factor *X*, *PAF_X_*, is defined as the following:

(1) $${\mathrm{PAF}}_{X}=\frac{P(Y=1)-P(Y=1|X=0)}{P(Y=1)},$$

where *Y* is the development of a disease of interest during a pre-specified interval, and *X* is a binary risk factor. It can be shown that *PAF_X_* also equals

(2) $$\begin{array}{l} {\mathrm{PAF}}_{X}=\\ P(X=1|Y=1)\frac{P(Y=1|X=1)/P(Y=1|X=0)-1}{P(Y=1|X=1)/P(Y=1|X=0)}. \end{array}$$

Given this relationship, it can be seen that *PAF_X_* is a function of the frequency of the risk factor and the relative risk (RR) associated with the risk factor. Let *X* be the count of risk alleles of the respective variant. With the assumption of Hardy-Weinberg Equilibrium, *X ∼ Bin*(2,*P_X_*), where *P_X_* is the risk allele frequency (RAF) in the general population. Under the model that X has additive effect on the log risk of Y such that the RR of developing the event Y per level increase in X is *e^β_x_^*, we can say

$$\begin{array}{c} P(Y=1)=P(Y=1|X=0){\left(1-P_{X}\right)}^{2}+2P(Y=1|X=0)\\ e^{\beta_{X}}P_{X}(1-P_{X})+P(Y=1|X=0)e^{2\beta_{X}}P_{X}^{2}. \end{array}$$

Similar to that derived earlier (Witte et al., 2014), *PAF_X_* is

(3) $$1-\frac{1}{{\left(1-P_{X}\right)}^{2}+2e^{\beta_{X}}P_{X}(1-P_{X})+e^{2\beta_{X}}P_{X}^{2}}.$$

For a set of *K* variants of interest, let the RAF of variant k (*k* = 1,…,K) be P*_X_k__*, and the RR of developing the event Y due to carrying each additional risk allele of this type be *e^β_k_^*, then the risk of event becomes

$$\begin{array}{l} P(Y=1)=\sum_{i_{1=0}}^{2}\cdots \sum_{i_{K=0}}^{2}P(Y=1|X_{1}=i_{1},\cdots ,X_{K}=i_{K})\\ P(X_{1}=i_{1},\cdots ,X_{K}=i_{K})\\ =P(Y=1|X_{1}=\cdots =X_{K}=0)\sum_{i_{1=0}}^{2}\cdots \sum_{i_{K=0}}^{2}e^{\sum_{k}\beta_{k}i_{k}}\\ P(X_{1}=i_{1},\cdots ,X_{K}=i_{K}). \end{array}$$

Similarly, the PAF of multiple variants is given by,

(4) $$\begin{array}{l} {\mathrm{PAF}}_{\left(X_{1},\cdots ,X_{K}\right)}=\\ 1-\frac{1}{\sum_{i_{1=1}}^{2}\cdots \sum_{i_{K=0}}^{2}e^{\sum_{k}\beta_{k}i_{k}}P(X_{1}=i_{1},\cdots ,X_{K}=i_{K})}. \end{array}$$

### Relationship Between PAF and Heritability *h*^2^ and $h_{L}^{2}$

To examine how the *PAF* and heritiablity are related, we start with a simple case that *Y* is a continuous trait (e.g., blood pressure, BMI), and then extend it to a binary trait. Let *Y* be a non-negative continuous trait value and *X* be a binary or a quantitative genetic exposure variable that is positively associated with *Y*. We define

$${\mathrm{PAF}}_{X}=\frac{E(Y)-E(Y|X=0)}{E(Y)}.$$

Assuming a linear regression model *E*(*Y*|*X*) = *β*_0_ + *β*_1_*X*, where *β*_0_ and *β*_1_ are regression coefficients, we can deduce that *PAF_X_* = $\frac{\beta_{1}E(X)}{E(Y)}$. For a continuous trait, the heritability attributed to one single variant is defined by the proportion of total variation of this trait across individuals in a given population that are explained by this genetic variant, i.e., *h*^2^ = $\frac{\beta_{1}^{2}\sigma_{X}^{2}}{\sigma_{Y}^{2}}$, where $\sigma_{X}^{2}$ and $\sigma_{Y}^{2}$ are the variances of *X* and *Y*, respectively. Thus, the heritability can be estimated by the coefficient of determination from a sample,

$${\hat{h}}^{2}=\frac{{\hat{\beta }}_{1}^{2}\sum_{i}{\left(X_{i}-\bar{X}\right)}^{2}}{\sum_{i}{\left(Y_{i}-\bar{Y}\right)}^{2}}.$$

Because ${\mathrm{PAF}}_{X}^{2}=\beta_{1}^{2}\frac{\sigma_{X}^{2}}{\sigma_{Y}^{2}}\frac{{\mathrm{CV}}_{Y}^{2}}{{\mathrm{CV}}_{X}^{2}}=h^{2}\frac{{\mathrm{CV}}_{Y}^{2}}{{\mathrm{CV}}_{X}^{2}}$, where *CV_Y_* and *CV_X_* are the coefficients of variations for *Y* and *X*, respectively, the estimate of *PAF_X_* is related to the estimate of heritaiblity as

(5) $$P\hat{A}F_{X}\approx \hat{h}\frac{\hat{C}V_{Y}}{\hat{C}V_{X}},$$

by replacing *h*^2^, *CV_X_*, and *CV_Y_* with their maximum likelihood estimates. It is to be noted that because a positive correlation between *Y* and *X* is assumed, 0 < *h* < 1.

Since the coefficient of variation measures the standard deviation of a particular variable after “standardizing” the variable to have a mean of one, equation (5) indicates that the PAF estimate equals the square root of the heritability estimate adjusted for the ratio in standard deviation between Y and X after standardization. Note that because PAF is on the same scale as *h* and they both are between 0 and 1, the PAF is much larger than *h*^2^.

In the situation to examine the genetic contribution attributed to multiple causal genetic variants, *X* is an *n* × *K* matrix for *n* individuals and *K* genetic variants and *β* is a vector of length *K* (excluding the intercept). As such,

$${\hat{h}}^{2}=\frac{V\hat{a}r(X\hat{\beta })}{V\hat{a}rY}\mathrm{and}\;P\hat{A}F_{X}=\frac{{\bar{X}}^{T}\hat{\beta }}{\bar{Y}}$$

where 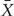 is a *K* × 1 vector of the average level of *X* over *n* individuals. Hence,

(6) $$P\hat{A}F_{X}\approx \hat{h}\frac{\hat{C}V_{Y}}{\hat{C}V_{X\hat{\beta }}},$$

where *ĈV_Xβ_* = $\frac{\sqrt{v\hat{a}r(X\beta )}}{{\bar{X}}^{T}\beta }$. Note that *ĥ* can be interpreted as the correlation between *Y* and *Ŷ* (the fitted value by the linear regression model) in both simple and multiple linear regressions. Similar to a single genetic variant, the square of *PÂF_X_* for multiple alleles equals the variability of *Y* that can be explained by *X* while adjusting for the ratio in variation between *Y* and *X*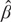 after “standardization”. Note that the heritability attributed to the whole-genome can also be estimated based on the normalized identity-by-state matrix (i.e., the empirical kinship matrix), *XX^T^*/*K*, where *X* here is whole-genome data and *K* is the dimension (Chen, 2014; Bulik-Sullivan et al., 2015).

In order to extend the relationship of PAF and heritability to a binary trait, first consider a log-linear model,

$$E(Y|Z)=e^{\delta_{0}+\delta Z},$$

where *Z* is the genotype value as defined earlier. Using a quadratic Taylor expansion at *δZ* = 0, this model can be approximated by the following linear regression model:

(7) $$E(Y|Z)\approx e^{\delta_{0}}+e^{\delta_{0}}\delta Z+\frac{e^{\delta_{0}}}{2}\delta^{2}Z^{2}=e^{\delta_{0}}+e^{\delta_{0}}(\delta Z+\frac{\delta^{2}}{2}Z^{2}).$$

Let *X* = (*Z,Z*^2^) and *β* = (*δ,δ*^2^/2) in equation (6), *PÂF_X_* = *ĥ*$\frac{\hat{C}V_{Y}}{\hat{C}V(e^{\delta_{0}}X\hat{\beta })}$, where *ĥ*^2^ is the heritability estimate on the observed scale for a binary outcome. Since *e*^*δ*_0_^ can be canceled out when computing the coefficient of variation of *e*^*δ*_0_^*Xβ*, finally we get *PÂF_X_* = *ĥ*$\frac{\hat{C}V_{Y}}{\hat{C}V_{X\hat{\beta }}}$.

Most frequently, a logistic regression model is used to model a binary trait,

$$E(Y|Z)=\frac{e^{\delta_{0}+\delta Z}}{1+e^{\delta_{0}+\delta Z}}.$$

A Taylor expansion at *δZ* = 0 of this model yields

(8) $$E(Y|Z)\approx \frac{e^{\delta_{0}}}{1+e^{\delta_{0}}}+\frac{e^{\delta_{0}}}{{\left(1+e^{\delta_{0}}\right)}^{2}}(\delta Z+\frac{1-e^{\delta_{0}}}{2(1+e^{\delta_{0}})}\delta^{2}Z^{2}).$$

Let *X* = (*Z,Z*^2^) and *β* = (*δ*, $\frac{1-e^{\delta_{0}}}{2(1+e^{\delta_{0}})}$*δ*^2^). The logistic regression model can also be approximated by the linear regression, so that equation (6) holds approximately. For logistic regression models, it is important to notice that the use of the Taylor expansion beyond the linear term requires the knowledge of *δ*_0_ (i.e, the baseline risk of event without the exposure). When the baseline risk of event is very small, equation (8) may be reduced to equation (7) as 1 + *e*^*δ*_0_^ ≈ 1 − *e*^*δ*_0_^ ≈ 1.

We have shown earlier that equation (6) indicates that the PAF estimate has a one-to-one relationship with the heritability estimate on the observed scale (*h*^2^). Since *h*^2^ is dependent on the prevalence of the trait in the population (*P_Y_*), it is often transformed to the underlying liability scale under the assumption of a classical liability threshold model (Falconer, 1965). It has been shown (Ge et al., 2017)

$$h_{L}^{2}={\mathrm{ch}}^{2},$$

where *c* = $\frac{P_{Y}(1-P_{Y})}{\upsilon^{2}}$ and υ is the height of the standard normal distribution at the threshold T such that *T* = Φ^−1^(1 − *P_Y_*). Thus, the PAF has a one-to-one correspondence with the heritability measured either on the observed or on the liability scale.

### Estimation of PAF Based on GWAS Summary Statistics

In the preceding sections, we have shown that there is a one-to-one relationship between the estimates of *PAF_X_*, *h*^2^ and $h_{L}^{2}$ for both continuous and binary traits. However, equation (6) may not be an optimal approach to estimate the *PAF_X_* because the use of Taylor series expansion can lead to a biased estimate especially for a logistic regression. Here, we propose a method to estimate the PAF using the GWAS summay statistics.

Consider a GWAS for a rare disease, which includes all the cases in a pre-defined population cohort and randomly selected controls from the cohort at either a 1:1 or 1:m (m > 1) ratio. As (1) the RAF for the population can be estimated based on the allele frequencies of cases and controls reported in the GWAS and (2) the RR associated with each additional allele of a genetic variant can be approximated for the estimated odds ratio (OR), we can estimate the *PAF_X_* using equation (3). Further, the 95% confidence interval for the *PAF_X_* can be estimated by a parametric bootstrap by sampling *β* from a normal distribution of *N*(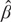
_*X*_,*ŝe*^2^(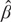
_*X*_)).

For a set of correlated genetic variants, their joint effect estimates on the risk of the event are required for estimating the PAF. However, often, only the marginal effect estimate of each individual variant being considered in the study is reported in the GWAS. While methods have been developed for linear regression models to estimate the joint effects of multiple alleles based on the marginal effect of each and the pairwise LD matrix obtained from the reference data (Pasaniuc and Price, 2017), such methods are currently not available for generalized linear regression models of binary outcomes. Here, we develop a Monte-Carlo method to estimate the joint effects of multiple alleles of interest using the GWAS summary statistics, from which we estimate the PAF contributed jointly by these alleles.

Our approach includes the following steps:

(1) Estimate the correlation between *Y* and *X_k_*. Let *ST_k_* be the score statistics of the assocation between *Y* and *X_k_*. The correlation between *Y* and *X_k_* (*k* = 1,...*K*)can then be estimated based on *ST_k_*, because
  $${\mathrm{ST}}_{k}=\frac{\sum_{i=1}^{N}x_{\mathrm{ik}}(y_{i}-\bar{y})}{\sqrt{\bar{y}(1-\bar{y})\sum_{k=1}^{N}{\left(x_{\mathrm{ik}}-{\bar{x}}_{k}\right)}^{2}}}\approx \mathrm{Corr}(Y,X_{k})*\sqrt{N},$$
  where *N* is the sample size of the case control study. It should be noted that ${\mathrm{ST}}_{k}^{2}$ approximately follows a Chi-square distribution. As the Chi-square statistics of Wald, score and liklihood ratio tests of a regression model, and Cochran-Amitage trend test are aymptotically equivalent, the p-value obtained from either test can be used to calculate the score statistic *ST_k_*.
(2) Simulate a large sample (for example, 100,000) of individual values of Y and (*X*_1_,⋅⋅⋅,*X_K_*) based on the estimated correlation between Y and each variant *X_k_* as well as the pairwise correlations between variants. The pairwise correlations between variants can be obtained from public available LD reference panels of the same ethnic population, such as 1000 Genome (Leisch and Andreas, 1998). The simulation of the sample is implemented using R package bindata. Based on the simulated data, (*β*_1_,⋅⋅⋅,*β_K_*) can then be estimated via a logistic regression of Y on all Xs simultaneously.
(3) Estimate *P*(*X*_1_,⋅⋅⋅,*X_K_*). To estimate the PAF, the joint distribution of all the variants within the population, *P*(*X*_1_,⋅⋅⋅,*X_K_*), needs to be estimated. Since cases were oversampled in case-control GWAS studies, *P*(*X*_1_,⋅ ⋅ ⋅,*X_K_*) can not be directly estimated from the simulated case-control data. Using the estimated RAF of *X_k_*, i.e., *P_X_k__*, we therefore empirically estimate the joint distribution of *X*_1_,⋅⋅⋅,*X_K_* in the population using *bindata*.
(4) Estimate the PAF based on equation (4).
(5) Estimate the 95% confidence interval (CI) of PAF using a parametric Bootstrap approach. Spefically, the standard error for the effect estimate can first be obtained based on the 95% CI reported in the GWAS result. That is, *sê*(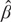
*_X_k__*) = ${\hat{\beta }}_{X_{k}}^{R}-{\hat{\beta }}_{X_{k}}^{L}$/(2^∗^1.96) so that we sample a large number (for example, 1000) of $\beta_{X_{k}}^{S}$ from *N*(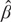
*_X_k__,sê*^2^(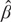
*_X_k__*)). Then, with each generated $\beta_{X_{k}}^{S}$, the score statistics ${\mathrm{ST}}_{k}^{S}\approx \frac{\beta_{X_{k}}^{S}}{s\hat{e}({\hat{\beta }}_{X_{k}})}$ is obtained, enabling the derivation of *Corr^S^*(*Y,X_k_*). Next, the *PAF^S^* is calculated using the steps 2–4 described above. The sample standard deviation of *PAF^S^* is used to construct the 95% CI for the PAF.

Based on the one-to-one correpondence between PAF and *h*^2^ and $h_{L}^{2}$ that we have established, the heritability along with its 95% CI contributed by multiple variants can be estimated. It is also important to point out that here we assume a rare disease, and thus we can approximate the RR associated with a risk allele by its OR. Further, when there are no other confounding and effect modification factors for the allele being examined, the following equation has been used to estimate the RR for a single binary variable when the disease is not very rare (McNutt et al., 1999; McNutt et al., 2003, 2014),

(9) $$\mathrm{RR}=\frac{\mathrm{OR}}{1-P(Y=1|X=0)+\mathrm{OR}\times P(Y=1|X=0)}.$$

When *X* is the count of a risk allele, however, equation (9) provides an approximation of RR because linearity in log of OR does not imply linearity in log of RR. The performance of the approximation varies by the magnitude of the OR and *P_X_*. Nonetheless, here we continue to use the above equation for the estimation of the RR associated with each allele, separately. In the following section we used simulation studies to examine the accuracy of this approximation for the purpose of estimating PAF.

## Simulation Studies

The purpose of the simulation studies is two-fold. First, we aim to examine the one-to-one correspondence that we have established between the estimates of PAF and *h*^2^ in a standard cohort study. Second, we aim to examine the performance of the proposed method for estimating the PAF based on the summary statistics obtained from the case-control GWAS. Based on the estimated PAF, we also estimated *h*^2^ and compared it with *h*^2^ obtained from the original cohort from which the case-control study was sampled.

To examine the relationship between the estimates of PAF and *h*^2^, a cohort of *N* = 100,000 was generated where the genetic exposure and disease association was defined based on a log linear regression model. We first examined the scenario with a single variant under an additive model. The disease incidence rate was set to be around 10%. We let the *P_x_* vary from 0.05 to 0.5 and the log of RR associated with *X* being 0.1, 0.2, and 0.3. These parameter values were chosen to cover a wide range of allele frequencies and allele-disease associations that are common in most GWAS studies, as well as in our examples. We next examined the scenario where three alleles were considered simutaneously, assuming that these alleles are either independent or with a moderate pairwise correlation of 0.2 or 0.3. For each senario, we repeated the simulaton1000 times, and reported the average and standard error of estimates of PAF, *h*^∗^ = *h*$\frac{{\mathrm{CV}}_{Y}}{{\mathrm{CV}}_{X\beta }}$ and *h*^2^. In our simulation, equation (3) was used to estimate the PAF of a single variant, where *P_X_* and *β_X_* were estimated from the simulated data, while equation (4) was used for multiple variants. We then repeated the simulation under a logistic regression model when the disease is rare, where the OR estimate was used to approximate RR. We also considered the situation when a dominant or recessive model is used for genetic variants.

Next, we simulated a case-control study, as it is the most common design in GWAS, to examine how the proposed method performs, especially when the disease is not very rare. To accomplish this, we first generated a cohort of *N* = 100,000 based on a logistic regression model with either a single variant or three variants under an additive model. The disease incidence rate in this set of simulations was set to be varying from ≤5% (very rare) and 5–10% (relatively common). Subjects of the study included all the cases in the cohort and an equal number of controls randomly selected from all the controls. To mimic the standard GWAS analysis, a logistic regression model was applied to each of the alleles separately to obtain the marginal estimate of OR, 95% CI, and *p*-value—the results typically reported in a GWAS. Based on these results, the proposed empirical method was used to estimate the *PAF_X_* and its 95% CI contributed by each of or all the alleles. It is also of interest to examine how well the method proposed in equation (9) is at correcting the potential bias in the estimated *PAF_X_* when the disease is not very rare. We reported the relative bias, defined as the difference between the average of the estimated PAF and the true PAF, and the coverage probability, defined as the proportion of times that the estimated confidence interval included the true PAF. The true PAF for each simulated cohort was calculated based on equation (1) where P(Y = 1) and P(Y = 1| *X* = 0) were pre-determined event rates for this cohort (not the GWAS subjects) and for those without the exposure, respectively. The average of *ĥ*^2^ and the bias of the average compared to the “true” *h*^2^ calculated directly from the cohort were also reported. As the computation is intensive, for each senario, the process was repeated 500 times. Finally, we examined the genetic variants that had either dominant or recessive effects using similar parameters.

## Applications

We applied the proposed method to estimate the PAF attributed to the genetic variants associated with lung cancer, as well as the heritability measures *h*^2^ and $h_{L}^{2}$. We first considered three independent susceptibility SNPs (rs7086803, rs9387478, and rs2395185) identified by our collaborative study of never-smoking Asian females. In the initial GWAS with 5,510 never-smoking female lung cancer cases and 4,544 controls from 14 studies of mainland China, South Korea, Japan and Singapore, Taiwan and Hong Kong, several new loci were identified to be significantly associated with lung cancer (Lan et al., 2012). The most promising variants were further genotyped in an additional 1,099 cases and 2,913 controls. Among them, rs7086803, rs9387478, and rs2395185 were independently validated. These three SNPs were on different genomic regions and were therefore independent from each other.

Then, we considered two correlated SNPs (rs2395185 and rs3817963). Both SNPs were at 6p21.3 locus close to the HLA-DRA region. The SNP, rs2395185, was significantly associated with lung adenocarcinoma in our collaborative GWAS; and rs3817963 was significantly associated with lung adenocarcinoma in a Japanese GWAS with a total of 6,029 cases and 13,535 controls (Shiraishi et al., 2012). The GWAS in the Japanese population included both genders. Since no SNP-gender and SNP-smoking interaction was found, we combined the results of these two studies to assess the PAF. These two SNPs are in a modest correlation in HapMap samples (*r*^2^ = 0.18 and 0.10 in Han Chinese and Japanese populations, respectively) (Lan et al., 2012). Thus, an averaged *r*^2^ of 0.14 was used in our calculations. As both studies were based on a case-control design, we used the proposed Monte-Carlo method to estimate the joint ORs accounting for their correlations. We further assumed that the joint distribution of (*X*_1_,⋅⋅⋅,*X_K_*) in the controls can be used to approximate the distribution in the general population. This assumption is considered to be appropriate as lung cancer among never-smoking Asian females is very rare [age-standardized incidence rate is 16.1/100,000 person-years with an average of 15 years of follow-up (Thun et al., 2008)].

## Results

### Simulation Studies

**Table 1A** shows the relationship between the estimates of PAF and heritability on the observed scale attributed to one genetic variant under a log-linear regression model. To estimate the PAF, a log-linear regression model was used to estimate the RR associated with the risk allele. As expected, we can see that both the estimates of PAF and *h*^2^ increase as the *P_x_* and RR increase. More importantly, **Table 1A** indicates that both the mean and the variation estimates of PAF and *h*^∗^ = *h*$\frac{{\mathrm{CV}}_{Y}}{{\mathrm{CV}}_{X\beta }}$ are in a very good agreement for most scenarios. As *P_x_* and RR increase, the estimates of PAF tend to be slightly smaller than the estimates of *h*^∗^. **Table 1B** shows the relationship between the estimates of PAF and heritability on the observed scale attributed to one genetic variant under a logistic regression model. A logistic regression model was used to estimate the OR associated with the risk allele. Again, a very good agreement between the estimates of PAF and *h*^∗^ was observed. The results were very similar when the risk allele had a dominant or recessive effect and therefore not shown.

**Table 1A:** Relationship between the estimates of PAF and heritability on the observed scale attributed to one genetic variant under a log-linear regression model.

*P_X_*	$\beta_{1}^{1}$	Average of PÂF based on R̂R(se)	Average of *ĥ*$\frac{\hat{C}V_{Y}}{\hat{C}V_{X\hat{\beta }}}$(se)	Average of *ĥ*^2^
0.05	0.1	0.010 (0.003)	0.010 (0.003)	0.00013
	0.2	0.022 (0.003)	0.022 (0.003)	0.00053
	0.3	0.034 (0.003)	0.034 (0.003)	0.00131
0.1	0.1	0.021 (0.005)	0.022 (0.005)	0.00024
	0.2	0.043 (0.004)	0.043 (0.005)	0.00099
	0.3	0.067 (0.005)	0.068 (0.005)	0.00250
0.2	0.1	0.041 (0.007)	0.041 (0.007)	0.00041
	0.2	0.083 (0.006)	0.085 (0.007)	0.00177
	0.3	0.127 (0.006)	0.132 (0.007)	0.00448
0.3	0.1	0.060 (0.008)	0.061 (0.009)	0.00052
	0.2	0.121 (0.008)	0.125 (0.008)	0.00235
	0.3	0.181 (0.007)	0.191 (0.008)	0.00594
0.4	0.1	0.080 (0.010)	0.081 (0.011)	0.00061
	0.2	0.157 (0.009)	0.163 (0.010)	0.00271
	0.3	0.230 (0.008)	0.246 (0.010)	0.00682
0.5	0.1	0.097 (0.012)	0.100 (0.013)	0.00063
	0.2	0.189 (0.011)	0.200 (0.012)	0.00282
	0.3	0.276 (0.009)	0.299 (0.011)	0.00718

*^1^RR = exp(β_1_) under a log linear model.*

**Table 1B:** Relationship between the estimates of PAF and heritability on the observed scale attributed to one genetic variant under a logistic regression model.

*P_X_*	*δ*_1_^1^	Average of PÂF based on ÔR(se)	Average of *ĥ*$\frac{\hat{C}V_{Y}}{\hat{C}V_{X\hat{\delta }}}$(se)	Average of *ĥ*^2^
0.05	0.1	0.010 (0.005)	0.010 (0.005)	0.00006
	0.2	0.022 (0.005)	0.020 (0.005)	0.00021
	0.3	0.034 (0.005)	0.032 (0.005)	0.00052
0.1	0.1	0.021 (0.007)	0.020 (0.007)	0.00022
	0.2	0.042 (0.007)	0.040 (0.007)	0.00039
	0.3	0.067 (0.007)	0.063 (0.007)	0.00098
0.2	0.1	0.042 (0.011)	0.040 (0.010)	0.00017
	0.2	0.083 (0.010)	0.080 (0.010)	0.00070
	0.3	0.127 (0.010)	0.122 (0.010)	0.00173
0.3	0.1	0.060 (0.013)	0.058 (0.013)	0.00022
	0.2	0.121 (0.012)	0.118 (0.012)	0.00093
	0.3	0.181 (0.012)	0.179 (0.012)	0.00227
0.4	0.1	0.080 (0.016)	0.077 (0.015)	0.00025
	0.2	0.157 (0.015)	0.154 (0.014)	0.00106
	0.3	0.230 (0.014)	0.231 (0.014)	0.00259
0.5	0.1	0.097 (0.019)	0.095 (0.019)	0.00026
	0.2	0.189 (0.017)	0.187 (0.017)	0.00109
	0.3	0.275 (0.015)	0.279 (0.016)	0.00267

*^1^OR=exp(δ_1_) under a logistic model.*

**Tables 2A,B** show the results when three genetic variants were considered simultaneously, with or without correlations. Again, a close agreement was observed between the PAF and *h*^∗^ estimates for both loglinear (**Table 2A**) and logistic regression models (**Table 2B**). The average estimate of PAF tended to be a bit smaller than that of the estimates of *h*^∗^ when *βX* or *δX* is high. This descrepancy is likely due to the inaccuracy of the Taylor expansion.

**Table 2A:** Relationship between PAF and heritability on the observed scale attributed to three genetic variants under a log-linear regression model.

*Cor*(*X*_1_,*X*_2_,*X*_3_)	(*P*_*X*_1__,*P*_*X*_2__,*P*_*X*_3__)	(*β*_1_,*β*_2_,*β*_3_)	Average of PÂF based on R̂R(se)	Average of *ĥ*$\frac{\hat{C}V_{Y}}{\hat{C}V_{X\hat{\beta }}}$(se)	Average of *ĥ*^2^
0.0	(0.1,0.1,0.1)	(0.1,0.1,0.1)	0.061 (0.007)	0.061 (0.007)	0.00073
		(0.3,0.2,0.1)	0.125 (0.007)	0.127 (0.007)	0.00408
	(0.1,0.2,0.3)	(0.1,0.1,0.1)	0.117 (0.010)	0.118 (0.011)	0.00127
		(0.3,0.2,0.1)	0.196 (0.009)	0.202 (0.010)	0.00571
	(0.2,0.3,0.4)	(0.1,0.1,0.1)	0.170 (0.013)	0.172 (0.013)	0.00176
		(0.3,0.2,0.1)	0.294 (0.010)	0.308 (0.016)	0.00970
	(0.3,0.4,0.5)	(0.1,0.1,0.1)	0.219 (0.014)	0.223 (0.015)	0.00215
		(0.3,0.2,0.1)	0.375 (0.010)	0.400 (0.012)	0.01308
0.2	(0.1,0.1,0.1)	(0.1,0.1,0.1)	0.062 (0.006)	0.062 (0.006)	0.00107
		(0.3,0.2,0.1)	0.129 (0.006)	0.132 (0.006)	0.00589
	(0.1,0.2,0.3)	(0.1,0.1,0.1)	0.119 (0.009)	0.120 (0.009)	0.00183
		(0.3,0.2,0.1)	0.201 (0.008)	0.209 (0.009)	0.00849
	(0.2,0.3,0.4)	(0.1,0.1,0.1)	0.172 (0.010)	0.174 (0.011)	0.00253
		(0.3,0.2,0.1)	0.300 (0.008)	0.316 (0.010)	0.01384
	(0.3,0.4,0.5)	(0.1,0.1,0.1)	0.222 (0.013)	0.226 (0.013)	0.00303
		(0.3,0.2,0.1)	0.382 (0.009)	0.408 (0.011)	0.01791

**Table 2B:** Relationship between PAF and heritability on the observed scale attributed to three genetic variants under a logistic regression model.

*Cor*(*X*_1_,*X*_2_,*X*_3_)	(*P*_*X*_1__,*P*_*X*_2__,*P*_*X*_3__)	(*δ*_1_,*δ*_2_,*δ*_3_)	Average of PÂF based on ÔR(se)	Average of *ĥ*$\frac{\hat{C}V_{Y}}{\hat{C}V_{X\hat{\delta }}}$(se)	Average of *ĥ*^2^
0.0	(0.1,0.1,0.1)	(0.1,0.1,0.1)	0.061 (0.012)	0.060 (0.011)	0.00031
		(0.3,0.2,0.1)	0.125 (0.011)	0.125 (0.010)	0.00158
	(0.1,0.2,0.3)	(0.1,0.1,0.1)	0.118 (0.017)	0.117 (0.016)	0.00053
		(0.3,0.2,0.1)	0.196 (0.014)	0.204 (0.014)	0.00217
	(0.2,0.3,0.4)	(0.1,0.1,0.1)	0.170 (0.019)	0.173 (0.018)	0.00070
		(0.3,0.2,0.1)	0.293 (0.017)	0.317 (0.016)	0.00350
	(0.3,0.4,0.5)	(0.1,0.1,0.1)	0.218 (0.023)	0.227 (0.022)	0.00083
		(0.3,0.2,0.1)	0.376 (0.016)	0.423 (0.016)	0.00459
0.3	(0.1,0.1,0.1)	(0.1,0.1,0.1)	0.063 (0.009)	0.062 (0.009)	0.00052
		(0.3,0.2,0.1)	0.132 (0.009)	0.132 (0.008)	0.00262
	(0.1,0.2,0.3)	(0.1,0.1,0.1)	0.120 (0.014)	0.121 (0.013)	0.00084
		(0.3,0.2,0.1)	0.205 (0.012)	0.215 (0.012)	0.00371
	(0.2,0.3,0.4)	(0.1,0.1,0.1)	0.173 (0.016)	0.176 (0.015)	0.00114
		(0.3,0.2,0.1)	0.302 (0.014)	0.326 (0.013)	0.00564
	(0.3,0.4,0.5)	(0.1,0.1,0.1)	0.222 (0.019)	0.230 (0.018)	0.00134
		(0.3,0.2,0.1)	0.386 (0.014)	0.428 (0.013)	0.00707

In the second set of simulations of a GWAS case-control study, the results for a single variant are summarized in **Table 3**. This **Table 3** shows that when the event rate was very rare (<5%), the bias of the PAF estimate was small and its 95% CI was close to its nominal coverage. However, when the disease event rate was between 5 and 10%, the PAF estimate based on ORs tended to be inflated up to 7%. This is most likely due to the overestimation of RR by directly using OR when the disease is relatively common. Based on the corrected OR estimate, the PAF estimate had a much smaller bias and its 95% CI was only slightly higher than the nominal coverge. The results for multiple variants are summarized in **Table 4**. The results were similar to those observed for a single variant, suggesting that the proposed Monte-Carlo method estimates the joint effect of multiple variants and its variation accurately.

**Table 3:** Estimation of PAF and h^2^ attributed to one genetic variant with and without the correction for OR.

P(Y = 1)	*P_X_*	*δ*_1_	Average of PÂF based on OR	Bias %^1^	Coverage %^2^	Average of PÂF based on corrected OR	Bias %	Coverage %	Average of *ĥ*^2^	Bias %
<5%	0.2	0.2	0.082	3.26	95.2	0.080	−0.43	96.7	0.00048	3.87
		0.3	0.126	3.64	97.2	0.121	−0.38	97.5	0.00115	3.08
	0.3	0.2	0.120	3.30	95.7	0.116	−0.34	96.9	0.00061	1.28
		0.3	0.181	3.60	95.6	0.175	−0.35	96.8	0.00146	−2.18
	0.5	0.2	0.189	2.38	95.2	0.182	−1.16	96.2	0.00072	−2.08
		0.3	0.275	3.14	93.6	0.267	−0.67	96.4	0.00163	−7.94
5–10%	0.2	0.2	0.082	5.37	94.7	0.077	−0.87	97.6	0.00080	9.15
		0.3	0.126	6.96	94.0	0.118	0.09	98.4	0.00194	6.56
	0.3	0.2	0.121	6.61	95.1	0.114	0.36	97.4	0.00103	7.65
		0.3	0.181	6.25	92.7	0.170	−0.46	97.9	0.00246	3.51
	0.5	0.2	0.191	6.45	92.0	0.179	0.34	96.3	0.00118	2.93
		0.3	0.275	5.89	89.4	0.259	−0.60	97.3	0.00275	−2.12

*^1^The relative bias is calculated as (estimated parameter-true parameter)/true parameter × 100%. ^2^The coverage probability is the proportion of the estimated 95% confidence intervals containing the true value.*

**Table 4:** Estimation of PAF and h^2^ attributed to three genetic variants with and without the correction for OR.

P(Y = 1)	(*δ*_1_,*δ*_2_,*δ*_3_)	*δ*_1_		*δ*_2_		*δ*_3_		*Average of* PÂF *based on OR*		*Average of* PÂF *based on corrected OR*		*Average of ĥ*^2^	% bias
		Bias %	Cov^1^ %	Bias %	Cov %	Bias %	Cov %	Bias %	Cov %	Bias %	Cov %		
<5%	(0.1,0.1,0.1)	3.97	99.4	−1.51	99.8	−0.01	99.2	4.56	99.8	0.51	99.8	0.000409	5.80
	(0.3,0.2,0.1)	4.42	99.2	1.71	99.4	1.78	99.2	6.80	97.6	2.35	99.4	0.001655	−5.91
5–10%	(0.1,0.1,0.1)	1.74	99.4	2.46	100.0	−3.68	99.8	5.95	99.4	−2.06	99.0	0.000815	7.53
	(0.3,0.2,0.1)	2.74	100.0	3.00	99.6	1.07	99.8	9.73	95.4	0.79	99.6	0.003258	−2.06

*^1^coverage.*

### Applications

**Table 5** shows the estimates of the PAF and heritability attributed to individual variants (rs7086803, rs9387478, and rs2395185 for overall lung cancer and rs381796 for lung adenocarcinoma). As expected, the estimated PAF (10–16%) was much larger than the corresponding estimate of heritability on the observed scale (2.69e-05 to 6.02e-05) as well as on the liability scale (0.0005–0.0015). It is interesting to note that the rank of estimated PAF was not necessarily consistent with the rank of heritability. The estimate of PAF of rs9387478 was the highest, while the estimate of heritability of rs7086803 was the highest. This is due to the fact that PAF is a function of both heritability and CV of the variant. **Table 6** showed that for lung cancer, three variants (rs7086803, rs9387478, and rs2395185) jointly contributed to 33.6% of the disease, and explained 0.03% of variability on the observed scale and 1.4% on the liability scale, respectively. For lung adenocarcinoma, two variants (rs2395185 and rs3817963) in the 6p21.3 regions together contributed to 14.8% of the disease, and explained 0.01% of variability on the observed scale and 0.6% on the liability scale, respectively.

**Table 5:** Estimates of PAF and heritability and 95% CI contributed by individual variants significantly associated with lung cancer in two GWAS conducted in Asian populations.

Variant^1^	*P*_*X*_^1^	OR	PÂF	*ĥ*^2^	${\hat{h}}_{\text{L}}^{2}$
rs7086803	0.27	1.28 (1.21,1.35)	0.136 (0.104,0.165)	6.02e-05 (3.57e-05,8.93e-05)	0.0015
rs9387478	0.50	0.85 (0.81, 0.90)^2^	0.156 (0.102,0.199)	2.93e-05 (1.27e-05,4.79e-05)	0.0005
rs2395185	0.36	1.17 (1.11,1.23)	0.111 (0.074,0.146)	2.69e-05 (1.20e-05,4.65e-05)	0.0005
rs3817963	0.32	1.18 (1.12.1.24)	0.105 (0.072,0.152)	2.88e-05 (1.35e-05,4.86e-05)	0.0006

*^1^The calculation of PAF and heritability of rs7086803, rs9387478, and rs2395185 were based on the first GWAS of N = 7421 never-smoking female lung cancer cases and 6512 controls drawn from 14 studies from mainland China, South Korea, Japan and Singapore, Taiwan and Hong Kong; the calculation of PAF and heritability of rs3817963 were based on the second GWAS of lung carcinoma conducted in a Japanese population with a total of 6,029 cases and 13,535 controls. ^2^The reciprocal of the OR was used to calculate PAF.*

**Table 6:** Estimates of PAF and heritability and 95% CI contributed by multiple variants significantly associated with lung cancer in two GWAS conducted in Asian populations.

Variants	PÂF	*ĥ*^2^	${\hat{h}}_{\text{L}}^{2}$
rs7086803,rs9387478,rs2395185^1^	0.336 (0.288,0.383)	0.000329 (0.00025,0.00043)	0.0140
rs2395185, rs3817963^2^	0.148 (0.110,0.189)	0.000144 (0.00008,0.00023)	0.0061

*^1^These three alleles are expected to be independent because rs7086803 is on a different chromosome with rs9387478 and rs2395185. rs9387478 and rs2395185 are both on chromosome 6 but at different regions. ^2^rs2395185 and rs3817963 are both at 6p21.3 locus, the r^2^ between these two variants in Han Chinese and Japanese HapMap samples was 0.18 and 0.10, respectively. An average r^2^ of 0.14 is used here. The estimated OR for lung adenocarcinoma associated with rs2398185 is 1.20 (1.12,1.28).*

## Discussion

In this paper, we examined the relationship between PAF and heritability, the two measures of genetic contribution to disease risk. Our results showed that for a given variant or a set of variants, the PAF is a one-to-one function of heritability. We also proposed a Monte-Carlo estimation procedure to estimate the PAF of multiple correlated risk alleles based on the case-control GWAS summary statistics. Using simulations, we confirmed the established relationship between PAF and heritability and showed the proposed method to estimate that PAF performed well.

There is a concern in the literature that using PAF to measure the genetic contribution to a disease may be inappropriate since the PAF estimates are often an order of magnitude larger than other measures, such as heritability. However, based on the established one-to-one function between the PAF and heritability estimates, we expect that PAF is larger than heritability: not because PAF is inadequate, but because PAF is on the same scale of the square root of heritability. Further, the PAF is also affected by the ratio in coefficient of variation between a binary trait and the genetic variant(s) being examined. In general, the relative variability of a trait is larger than that of a genetic variant: the lower the event rate, the larger the ratio in their CVs. For example, for an event with 10% event rate, the ratio in coefficient of variation between Y and X is 2.12, 2.78, and 4.24 for *P_X_* = 0.2, 0.3, and 0.5, respectively, under an additive model; for a rare event with 1% event rate, these ratios are 7.04, 9.21, and 14.07, respectively. While heritability is an important measure to help understand to what extent the genetic variants explain population variation, PAF is also useful to measure the effects of the genetic variants on the mean risk of an event so that the impact on the overall event rate can be estimated if theoretical intervention on certain risk alleles are successful. Further, it is important to note that the ranks of these two measures for the same set of genetic variants do not necessarily agree. For example, the PAF estimate of a genetic variant associated with Crohn’s disease, for which the minor allele frequency was 0.07% and the RR associated with the minor allele was 0.42 (i.e., the minor allele had a protective effect), was 81%, while the heritability estimate on the liability scale was only 1.02% (Jostins et al., 2012; Witte et al., 2014). The small heritability estimate suggests that this variant contributes very little to the variability of the population risk because few people carry the minor allele. On the other hand, the large PAF estimate indicates a significant reduction in the average population risk if a successful intervention is available for a very large proportion of individuals carrying the common risk allele (about 99%). In summary, both these measures are useful as they describe the different aspects of the genetic contribution to disease risks.

It is important to point out that heritability does not imply causality, neither does PAF. In fact, the PAF does not add any new causal information to the RR (Greenland and Robins, 1988; Flegal et al., 2015). Unless the biologic mechanism is established, the PAF does not necessarily imply population “excess” risk attributed to the exposure. While the effect estimate of a genetic exposure may be more robust to confounding factors and reverse causality than environmental risk factors, the observed effects of genetic variants may result from LD as well as bias from population stratification. When the variant examined is not causal but in LD with the causal one, the heritability is likely to be underestimated because of the attenuation of the effect estimate (Visscher et al., 2010), and so is the PAF. Thus, the PAF also needs to be interpreted cautiously in genetic studies.

The overall PAF attributed to multiple alleles denoted by (*X*_1_,...,*X_K_*), when they are independent is *PAF* = 1 − $\prod_{k=1}^{K}$ (1 − *PAF_X_K__*), where *PAF_X_K__* is obtained based on the marginal OR of *X_k_* (Witte et al., 2014). However, when the risk alleles are positively correlated, the marginal OR of each individual risk allele is larger than the true OR as it includes a fraction of the effect of the other correlated risk alleles. Therefore, 1 − $\prod_{k=1}^{K}$ (1 − *PAF_X_K__*) can significantly overestimate the overall *PAF* (Rockhill et al., 1998). When alleles are correlated, the overall *PAF* should be approximated by $1-\prod_{k=1}^{K}(1-{\mathrm{PAF}}_{X_{k}}^{a}),$, where ${\mathrm{PAF}}_{X_{K}}^{a}$ is obtained based on the OR adjusted for other variants. In our example of lung adenocarcinoma, the marginal ORs associated with the two correlated risk alleles of rs2395185 and rs3817963 were 1.20 and 1.18, respectively, while the corresponding adjusted ORs were 1.13 and 1.10. As a result, the ${\mathrm{PAF}}_{X_{K}}^{a}$ estimates (0.087 and 0.061 for rs2395185 and rs3817963, respectively) were smaller than the PAF estimates based on marginal ORs (**Table 5**). The joint PAF based on the adjusted ORs can be approximated by 1-(1-0.087) (1-0.061) = 0.142, which is very close to what we obtained in **Table 6**.

In this paper, we developed an empirical method to estimate the joint effects of multiple alleles. As mentioned earlier, the joint effect estimates in a linear regression model can be mathematically obtained from the GWAS summary statistics of individual variants (Pasaniuc and Price, 2017). However, to our knowledge there is no existing method available for a logistic regression. The difficulty primarily results from the fact that in a logistic regression model the parameter estimation does not have a closed-form solution. Instead, it requires an iteratively reweighted least square algorithm. The proposed empirical Monte-Carlo method is conceptually straightforward, but is computationally intensive when the number of genetic variants is large. It is desirable in our future work to derive a numerical solution to estimate the joint effects from summary statistics of logistic and log linear regression models. This work is critical because not only can it simplify the computation and hence allow us to examine a large number of variants simultaneously but also can have other important applications, e.g., building a prediction model of disease risk on multiple genetic variants using the GWAS summary statistics.

Furthermore, our approach can be extended beyond the main effects of genetic variants to situations when gene-environment interactions exist [e.g., coal use as a risk exposure to lung cancer (Hosgood et al., 2015)] by examining the PAF separately for subjects with and without the environmental exposure. However, future research is warranted for the assessment of the overall PAF attributed by gene and environmental exposures simultaneously and when the environmental exposure is a continuous variable.

## Conclusion

In conclusion, we show that there is a one-to-one relationship between the estimates of PAF and heritability. Each of these two measures indicates a distinct and useful aspect of the genetic contribution to disease risk. We hope this paper will help to provide a more comprehensive understanding of PAF and foster a wider use of PAF in genetic studies.

## Author Contributions

TW and XX conceived of the presented idea, developed the theory, and performed the computations. HDH and QL verified the analytical methods. All authors discussed the results and contributed to the final manuscript.

## Conflict of Interest Statement

The authors declare that the research was conducted in the absence of any commercial or financial relationships that could be construed as a potential conflict of interest.
